# *catena*-Poly[[[tetra­aqua­cobalt(II)]-μ-4,4′-bipyridine-κ^2^ *N*:*N*′] bis­(perchlorate) 4,4′-bipyridine disolvate dihydrate]

**DOI:** 10.1107/S1600536808035125

**Published:** 2008-10-31

**Authors:** Joseph H. Nettleman, Robert L. LaDuca

**Affiliations:** a Lyman Briggs College, Department of Chemistry, Michigan State University, East Lansing, MI 48825, USA

## Abstract

In the title compound, {[Co(C_10_H_8_N_2_)(H_2_O)_4_](ClO_4_)_2_·2C_10_H_8_N_2_·2H_2_O}_*n*_, slightly distorted octa­hedrally coordinated Co^II^ ions situated on inversion centers are linked into polycationic chains through 4,4′-bipyridine tethering ligands. These are connected into supra­molecular layers by hydrogen bonding involving aqua ligands, perchlorate anions and uncoordinated water mol­ecules. A twofold inter­penetrated primitive cubic supra­molecular network is formed by the inter­action of pseudo-layers by hydrogen bonding between aqua ligands and unligated 4,4′-bipyridine mol­ecules.

## Related literature

For a review of coordination polymers containing 4,4′-bipyridine, see: Yaghi *et al.* (1998 ▶).
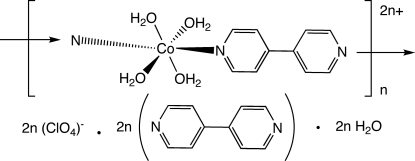


## Experimental

### 

#### Crystal data

[Co(C_10_H_8_N_2_)(H_2_O)_4_](ClO_4_)_2_·2C_10_H_8_N_2_·2H_2_O
*M* *_r_* = 834.48
Triclinic, 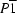

*a* = 8.9590 (17) Å
*b* = 10.846 (2) Å
*c* = 11.433 (2) Å
α = 64.290 (2)°
β = 71.747 (2)°
γ = 66.848 (2)°
*V* = 906.6 (3) Å^3^
*Z* = 1
Mo *K*α radiation
μ = 0.70 mm^−1^
*T* = 173 (2) K
0.35 × 0.30 × 0.25 mm

#### Data collection

Bruker SMART 1K diffractometer
Absorption correction: multi-scan (*SADABS*; Sheldrick, 1996 ▶) *T*_min_ = 0.793, *T*_max_ = 0.845
10254 measured reflections
4087 independent reflections
3754 reflections with *I* > 2σ(*I*)
*R*_int_ = 0.018

#### Refinement

*R*[*F* ^2^ > 2σ(*F* ^2^)] = 0.030
*wR*(*F* ^2^) = 0.085
*S* = 1.06
4087 reflections
259 parameters
9 restraints
H atoms treated by a mixture of independent and constrained refinement
Δρ_max_ = 0.35 e Å^−3^
Δρ_min_ = −0.32 e Å^−3^

### 

Data collection: *SMART* (Bruker, 2003 ▶); cell refinement: *SAINT-Plus* (Bruker, 2003 ▶); data reduction: *SAINT-Plus*; program(s) used to solve structure: *SHELXS97* (Sheldrick, 2008 ▶); program(s) used to refine structure: *SHELXL97* (Sheldrick, 2008 ▶); molecular graphics: *Crystal Maker* (Palmer, 2007 ▶); software used to prepare material for publication: *SHELXL97*.

## Supplementary Material

Crystal structure: contains datablocks I, global. DOI: 10.1107/S1600536808035125/ng2505sup1.cif

Structure factors: contains datablocks I. DOI: 10.1107/S1600536808035125/ng2505Isup2.hkl

Additional supplementary materials:  crystallographic information; 3D view; checkCIF report

## Figures and Tables

**Table 1:** Hydrogen-bond geometry (Å, °)

*D*—H⋯*A*	*D*—H	H⋯*A*	*D*⋯*A*	*D*—H⋯*A*
O1—H1*A*⋯O1*W*	0.856 (15)	1.886 (16)	2.7406 (19)	176 (2)
O1—H1*B*⋯N7^i^	0.852 (15)	1.943 (16)	2.7744 (18)	165 (2)
O1*W*—H1*WA*⋯O3^ii^	0.868 (16)	2.079 (17)	2.924 (2)	164 (2)
O1*W*—H1*WB*⋯O6	0.881 (16)	2.192 (16)	3.070 (3)	174 (2)
O2—H2*A*⋯N6^iii^	0.883 (15)	1.826 (15)	2.7058 (19)	174.1 (19)
O2—H2*B*⋯O3^iv^	0.849 (15)	2.212 (17)	2.957 (2)	146.5 (18)

Symmetry codes: (i) 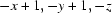; (ii) 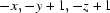; (iii) 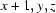; (iv) 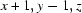.

**Fig. 1.**
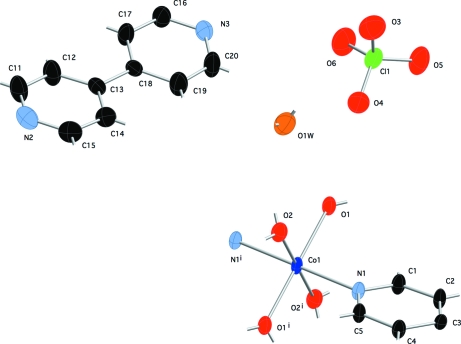
Asymmetric unit of the title compound, showing 50% probability ellipsoids and atom numbering scheme. Hydrogen atom positions are shown as gray sticks. Color codes: dark blue Co, light blue N, red O, orange O within unligated water molecule, black C, green Cl.

**Fig. 2.**
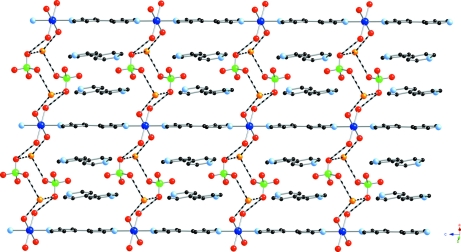
A single supramolecular layer in the title compound, featuring one-dimensional [Co(H2O)4(4,4-bipyridine)]n chains. Hydrogen bonding is indicated as dashed lines.

**Fig. 3.**
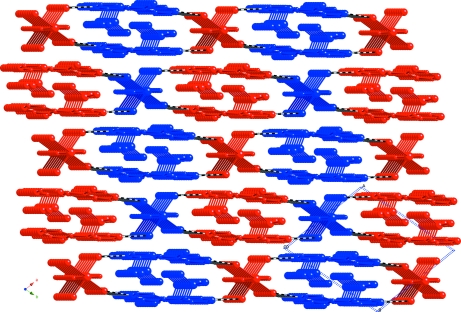
Packing diagram illustrating the ABAB layer stacking pattern, which forms the 3-D crystal structure of the title compound through hydrogen bonding between ligated water molecules and uncoordinated 4,4'-bipyridine molecules.

**Fig. 4.**
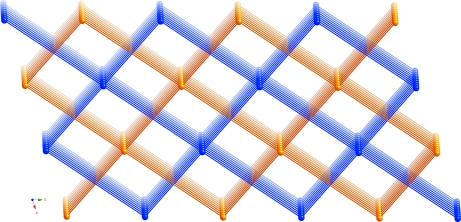
Schematic perspective of the twofold interpenetrated primitive cubic supramolecular network of the title compound.

## supplementary crystallographic information

### Comment

The dipodal tethering ligand 4,4'-bipyridine has proven extremely advantageous for the construction of coordination polymer solids (Yaghi *et al.*, 1998). In an attempt to prepare a divalent cobalt coordination polymer incorporating both 2-methylglutarate and 4,4'-bipyridine, yellow block crystals of the title compound were obtained.

The asymmetric unit of the title compound contains a cobalt atom on a crystallographic inversion center, two aqua ligands, one-half of a 4,4'-bipyridine ligand, one uncoordinated perchlorate anion, one unligated 4,4'-bipyridine molecule and one water molecule of crystallization (Figure 1).

Tethering 4,4'-bipyridine ligands connect the Co^II^ ions into one-dimensional cationic {[Co(H_2_O)_4_(C_10_H_8_N_2_)]_n_^2n+^ chain motifs that are oriented parallel to the *c* crystal direction. The Co···Co through-ligand contact distance is 11.433 (2) Å. These chains are connected into pseudolayer patterns by hydrogen-bonding mechanisms involving the aqua ligands, perchlorate anions, and water molecules of crystallization (Figure 2), which lie parallel to the (1 1 0) crystal planes. Unligated 4,4'-bipyridine molecules project axially into and out of the apertures in each pseudolayer.

In turn, the pseudolayers stack in an *AB* arrangement, and interact with their next nearest neighbors by hydrogen-bonding donation from aqua ligands to the uncoordinated 4,4'-bipyridine molecules to form the three-dimensional structure of the title compound (Figure 3). As a result a twofold interpenetrated primitive cubic supramolecular network can be invoked (Figure 4).

### Experimental

All chemicals were obtained commercially. Cobalt perchlorate hexahydrate (135 mg, 0.37 mmol), 2-methylglutaric acid (59 mg, 0.37 mmol) and 4,4'-bipyridine (116 mg, 0.74 mmol) were placed into 10 ml H_2_O in a 23 ml Teflon-lined Parr acid digestion bomb. The bomb was heated at 120° C for 48 h and was then allowed to cool to 25° C. Yellow-orange crystals of the title compound were obtained along with a reddish amorphous solid.

### Refinement

All H atoms bound to C atoms were placed in calculated positions, with C—H = 0.95 Å and refined in riding mode with *U*_iso_ = 1.2*U*_eq_(C). All H atoms bound to O atoms were found *via* Fourier difference map, restrained with O—H = 0.89 Å o, and refined with *U*_iso_=1.2*U*_eq_(O).

### Crystal data

**Table:**

[Co(C_10_H_8_N_2_)(H_2_O)_4_](ClO_4_)_2_·2C_10_H_8_N_2_·2H_2_O	*Z* = 1
*M**_r_* = 834.48	*F*(000) = 431
Triclinic, *P*1	*D*_x_ = 1.528 Mg m^−^^3^
*a* = 8.9590 (17) Å	Mo *K*α radiation, λ = 0.71073 Å
*b* = 10.846 (2) Å	Cell parameters from 10254 reflections
*c* = 11.433 (2) Å	θ = 2.0–28.2°
α = 64.290 (2)°	µ = 0.70 mm^−^^1^
β = 71.747 (2)°	*T* = 173 K
γ = 66.848 (2)°	Block, yellow
*V* = 906.6 (3) Å^3^	0.35 × 0.30 × 0.25 mm

### Data collection

**Table:**

Bruker SMART 1K diffractometer	4087 independent reflections
Radiation source: fine-focus sealed tube	3754 reflections with *I* > 2σ(*I*)
graphite	*R*_int_ = 0.018
ω scans	θ_max_ = 28.2°, θ_min_ = 2.0°
Absorption correction: multi-scan (SADABS; Sheldrick, 1996)	*h* = −11→11
*T*_min_ = 0.793, *T*_max_ = 0.845	*k* = −14→14
10254 measured reflections	*l* = −15→15

### Refinement

**Table:**

Refinement on *F*^2^	Primary atom site location: structure-invariant direct methods
Least-squares matrix: full	Secondary atom site location: difference Fourier map
*R*[*F*^2^ > 2σ(*F*^2^)] = 0.030	Hydrogen site location: inferred from neighbouring sites
*wR*(*F*^2^) = 0.085	H atoms treated by a mixture of independent and constrained refinement
*S* = 1.06	*w* = 1/[σ^2^(*F*_o_^2^) + (0.0438*P*)^2^ + 0.4101*P*] where *P* = (*F*_o_^2^ + 2*F*_c_^2^)/3
4087 reflections	(Δ/σ)_max_ < 0.001
259 parameters	Δρ_max_ = 0.35 e Å^−^^3^
9 restraints	Δρ_min_ = −0.32 e Å^−^^3^

### Special details

**Table:**

Geometry. All e.s.d.'s (except the e.s.d. in the dihedral angle between two l.s. planes) are estimated using the full covariance matrix. The cell e.s.d.'s are taken into account individually in the estimation of e.s.d.'s in distances, angles and torsion angles; correlations between e.s.d.'s in cell parameters are only used when they are defined by crystal symmetry. An approximate (isotropic) treatment of cell e.s.d.'s is used for estimating e.s.d.'s involving l.s. planes.
Refinement. Refinement of *F*^2^ against ALL reflections. The weighted *R*-factor *wR* and goodness of fit *S* are based on *F*^2^, conventional *R*-factors *R* are based on *F*, with *F* set to zero for negative *F*^2^. The threshold expression of *F*^2^ > σ(*F*^2^) is used only for calculating *R*-factors(gt) *etc*. and is not relevant to the choice of reflections for refinement. *R*-factors based on *F*^2^ are statistically about twice as large as those based on *F*, and *R*- factors based on ALL data will be even larger.

### Fractional atomic coordinates and isotropic or equivalent isotropic displacement parameters (Å^2^)

**Table:**

	*x*	*y*	*z*	*U*_iso_*/*U*_eq_	
Co1	0.5000	0.0000	0.5000	0.02019 (9)	
Cl1	0.08313 (6)	0.68822 (5)	0.28417 (4)	0.03844 (12)	
O1	0.49177 (16)	0.21628 (12)	0.41627 (11)	0.0331 (3)	
H1A	0.432 (2)	0.272 (2)	0.4590 (19)	0.040*	
H1B	0.527 (2)	0.269 (2)	0.3396 (16)	0.040*	
O1W	0.31208 (19)	0.40133 (17)	0.54939 (15)	0.0471 (3)	
H1WA	0.233 (3)	0.366 (2)	0.602 (2)	0.057*	
H1WB	0.258 (3)	0.4859 (19)	0.499 (2)	0.057*	
O2	0.75905 (14)	−0.06554 (13)	0.46036 (11)	0.0291 (2)	
H2A	0.818 (2)	−0.0077 (19)	0.4057 (18)	0.035*	
H2B	0.809 (2)	−0.1404 (17)	0.4406 (19)	0.035*	
O3	−0.0888 (2)	0.76945 (18)	0.28167 (18)	0.0592 (4)	
O4	0.1008 (2)	0.53921 (16)	0.32135 (15)	0.0576 (4)	
O5	0.1756 (2)	0.73950 (19)	0.15629 (15)	0.0586 (4)	
O6	0.1383 (3)	0.7065 (2)	0.37918 (17)	0.0661 (5)	
N1	0.49875 (15)	−0.00436 (13)	0.31409 (11)	0.0222 (2)	
N6	−0.05017 (19)	0.09941 (17)	0.28287 (17)	0.0413 (4)	
N7	0.3813 (2)	0.59027 (15)	−0.19049 (14)	0.0368 (3)	
C1	0.5839 (2)	0.06703 (17)	0.20323 (14)	0.0269 (3)	
H1	0.6438	0.1163	0.2098	0.032*	
C2	0.5878 (2)	0.07151 (17)	0.07951 (14)	0.0263 (3)	
H2	0.6488	0.1230	0.0057	0.032*	
C3	0.49982 (17)	−0.00143 (15)	0.06583 (13)	0.0195 (3)	
C4	0.41216 (18)	−0.07678 (16)	0.18123 (14)	0.0240 (3)	
H4	0.3528	−0.1284	0.1777	0.029*	
C5	0.41367 (19)	−0.07458 (16)	0.30132 (14)	0.0246 (3)	
H5	0.3527	−0.1242	0.3767	0.030*	
C11	−0.0014 (3)	0.1789 (2)	0.3167 (2)	0.0486 (5)	
H11	−0.0296	0.1723	0.4043	0.058*	
C12	0.0890 (3)	0.2709 (2)	0.22857 (18)	0.0462 (5)	
H12	0.1211	0.3231	0.2576	0.055*	
C13	0.13149 (19)	0.28494 (16)	0.09666 (16)	0.0292 (3)	
C14	0.0826 (2)	0.20049 (19)	0.06118 (19)	0.0358 (4)	
H14	0.1089	0.2049	−0.0257	0.043*	
C15	−0.0056 (2)	0.1098 (2)	0.1566 (2)	0.0413 (4)	
H15	−0.0355	0.0530	0.1315	0.050*	
C16	0.3327 (3)	0.5801 (2)	−0.06572 (18)	0.0414 (4)	
H16	0.3518	0.6430	−0.0410	0.050*	
C17	0.2551 (3)	0.4816 (2)	0.03043 (17)	0.0404 (4)	
H17	0.2253	0.4787	0.1170	0.048*	
C18	0.22204 (19)	0.38758 (16)	−0.00284 (15)	0.0279 (3)	
C19	0.2767 (3)	0.3953 (2)	−0.13273 (19)	0.0515 (5)	
H19	0.2608	0.3330	−0.1603	0.062*	
C20	0.3555 (3)	0.4965 (2)	−0.22154 (19)	0.0544 (6)	
H20	0.3924	0.4988	−0.3079	0.065*	

### Atomic displacement parameters (Å^2^)

**Table:**

	*U*^11^	*U*^22^	*U*^33^	*U*^12^	*U*^13^	*U*^23^
Co1	0.02731 (15)	0.02675 (15)	0.01189 (13)	−0.01560 (11)	−0.00041 (10)	−0.00699 (10)
Cl1	0.0476 (3)	0.0431 (2)	0.0316 (2)	−0.0248 (2)	0.00596 (17)	−0.01835 (18)
O1	0.0526 (7)	0.0292 (6)	0.0185 (5)	−0.0218 (5)	0.0047 (5)	−0.0082 (4)
O1W	0.0497 (8)	0.0490 (8)	0.0415 (8)	−0.0157 (7)	0.0008 (6)	−0.0204 (6)
O2	0.0291 (6)	0.0366 (6)	0.0254 (5)	−0.0162 (5)	0.0013 (4)	−0.0126 (5)
O3	0.0490 (9)	0.0616 (10)	0.0690 (11)	−0.0162 (7)	−0.0001 (8)	−0.0324 (8)
O4	0.0855 (12)	0.0422 (8)	0.0454 (8)	−0.0285 (8)	0.0030 (8)	−0.0176 (6)
O5	0.0724 (10)	0.0718 (10)	0.0419 (8)	−0.0498 (9)	0.0185 (7)	−0.0240 (7)
O6	0.0940 (13)	0.0762 (11)	0.0512 (10)	−0.0387 (10)	−0.0143 (9)	−0.0300 (9)
N1	0.0272 (6)	0.0290 (6)	0.0148 (5)	−0.0139 (5)	−0.0012 (4)	−0.0084 (5)
N6	0.0317 (7)	0.0383 (8)	0.0466 (9)	−0.0179 (6)	−0.0048 (6)	−0.0035 (7)
N7	0.0455 (8)	0.0305 (7)	0.0288 (7)	−0.0171 (6)	0.0015 (6)	−0.0062 (6)
C1	0.0365 (8)	0.0369 (8)	0.0179 (7)	−0.0237 (7)	−0.0007 (6)	−0.0104 (6)
C2	0.0355 (8)	0.0357 (8)	0.0150 (6)	−0.0230 (7)	0.0013 (5)	−0.0083 (6)
C3	0.0222 (6)	0.0232 (6)	0.0149 (6)	−0.0080 (5)	−0.0027 (5)	−0.0077 (5)
C4	0.0295 (7)	0.0316 (7)	0.0175 (7)	−0.0175 (6)	−0.0021 (5)	−0.0086 (5)
C5	0.0311 (7)	0.0325 (7)	0.0152 (6)	−0.0188 (6)	−0.0002 (5)	−0.0073 (5)
C11	0.0590 (12)	0.0563 (12)	0.0314 (9)	−0.0358 (10)	−0.0022 (8)	−0.0037 (8)
C12	0.0651 (13)	0.0516 (11)	0.0308 (9)	−0.0385 (10)	−0.0033 (8)	−0.0073 (8)
C13	0.0276 (7)	0.0241 (7)	0.0303 (8)	−0.0082 (6)	−0.0051 (6)	−0.0044 (6)
C14	0.0310 (8)	0.0381 (9)	0.0398 (9)	−0.0132 (7)	−0.0030 (7)	−0.0147 (7)
C15	0.0327 (9)	0.0415 (10)	0.0537 (11)	−0.0185 (8)	−0.0027 (8)	−0.0170 (8)
C16	0.0592 (12)	0.0392 (9)	0.0326 (9)	−0.0292 (9)	0.0017 (8)	−0.0125 (7)
C17	0.0576 (11)	0.0425 (10)	0.0258 (8)	−0.0297 (9)	0.0053 (7)	−0.0119 (7)
C18	0.0285 (7)	0.0231 (7)	0.0271 (8)	−0.0075 (6)	−0.0041 (6)	−0.0053 (6)
C19	0.0893 (16)	0.0470 (11)	0.0311 (9)	−0.0427 (11)	0.0009 (10)	−0.0131 (8)
C20	0.0916 (17)	0.0531 (12)	0.0263 (9)	−0.0436 (12)	0.0055 (10)	−0.0123 (8)

### Geometric parameters (Å, °)

**Table:**

Co1—O1^i^	2.0938 (12)	C2—C3	1.3950 (19)
Co1—O1	2.0938 (12)	C2—H2	0.9300
Co1—O2^i^	2.1024 (12)	C3—C4	1.3948 (19)
Co1—O2	2.1024 (12)	C3—C3^ii^	1.491 (3)
Co1—N1	2.1500 (12)	C4—C5	1.3878 (19)
Co1—N1^i^	2.1500 (12)	C4—H4	0.9300
Cl1—O5	1.4251 (15)	C5—H5	0.9300
Cl1—O4	1.4361 (15)	C11—C12	1.385 (3)
Cl1—O6	1.4365 (16)	C11—H11	0.9300
Cl1—O3	1.4450 (17)	C12—C13	1.389 (3)
O1—H1A	0.856 (15)	C12—H12	0.9300
O1—H1B	0.852 (15)	C13—C14	1.395 (2)
O1W—H1WA	0.868 (16)	C13—C18	1.492 (2)
O1W—H1WB	0.881 (16)	C14—C15	1.387 (3)
O2—H2A	0.883 (15)	C14—H14	0.9300
O2—H2B	0.849 (15)	C15—H15	0.9300
N1—C1	1.3399 (18)	C16—C17	1.385 (2)
N1—C5	1.3435 (18)	C16—H16	0.9300
N6—C11	1.333 (3)	C17—C18	1.384 (2)
N6—C15	1.337 (3)	C17—H17	0.9300
N7—C16	1.322 (2)	C18—C19	1.386 (3)
N7—C20	1.328 (3)	C19—C20	1.388 (3)
C1—C2	1.384 (2)	C19—H19	0.9300
C1—H1	0.9300	C20—H20	0.9300
			
O1^i^—Co1—O1	180.000 (1)	C4—C3—C2	116.62 (12)
O1^i^—Co1—O2^i^	91.24 (5)	C4—C3—C3^ii^	121.98 (15)
O1—Co1—O2^i^	88.76 (5)	C2—C3—C3^ii^	121.39 (15)
O1^i^—Co1—O2	88.76 (5)	C5—C4—C3	119.88 (13)
O1—Co1—O2	91.24 (5)	C5—C4—H4	120.1
O2^i^—Co1—O2	180.0	C3—C4—H4	120.1
O1^i^—Co1—N1	87.77 (5)	N1—C5—C4	123.30 (13)
O1—Co1—N1	92.23 (5)	N1—C5—H5	118.3
O2^i^—Co1—N1	90.66 (4)	C4—C5—H5	118.3
O2—Co1—N1	89.34 (4)	N6—C11—C12	123.61 (19)
O1^i^—Co1—N1^i^	92.23 (5)	N6—C11—H11	118.2
O1—Co1—N1^i^	87.77 (5)	C12—C11—H11	118.2
O2^i^—Co1—N1^i^	89.34 (4)	C11—C12—C13	119.77 (18)
O2—Co1—N1^i^	90.66 (4)	C11—C12—H12	120.1
N1—Co1—N1^i^	180.0	C13—C12—H12	120.1
O5—Cl1—O4	110.45 (9)	C12—C13—C14	116.80 (16)
O5—Cl1—O6	110.12 (11)	C12—C13—C18	121.64 (16)
O4—Cl1—O6	109.46 (11)	C14—C13—C18	121.55 (15)
O5—Cl1—O3	108.74 (11)	C15—C14—C13	119.38 (17)
O4—Cl1—O3	108.86 (11)	C15—C14—H14	120.3
O6—Cl1—O3	109.19 (11)	C13—C14—H14	120.3
Co1—O1—H1A	119.4 (14)	N6—C15—C14	123.67 (18)
Co1—O1—H1B	133.1 (14)	N6—C15—H15	118.2
H1A—O1—H1B	106.9 (18)	C14—C15—H15	118.2
H1WA—O1W—H1WB	102 (2)	N7—C16—C17	124.13 (17)
Co1—O2—H2A	123.8 (13)	N7—C16—H16	117.9
Co1—O2—H2B	118.8 (13)	C17—C16—H16	117.9
H2A—O2—H2B	101.8 (18)	C18—C17—C16	119.71 (16)
C1—N1—C5	116.71 (12)	C18—C17—H17	120.1
C1—N1—Co1	119.99 (9)	C16—C17—H17	120.1
C5—N1—Co1	123.29 (9)	C17—C18—C19	116.31 (15)
C11—N6—C15	116.74 (16)	C17—C18—C13	121.17 (15)
C16—N7—C20	116.24 (15)	C19—C18—C13	122.51 (16)
N1—C1—C2	123.68 (13)	C18—C19—C20	119.67 (18)
N1—C1—H1	118.2	C18—C19—H19	120.2
C2—C1—H1	118.2	C20—C19—H19	120.2
C1—C2—C3	119.79 (13)	N7—C20—C19	123.85 (18)
C1—C2—H2	120.1	N7—C20—H20	118.1
C3—C2—H2	120.1	C19—C20—H20	118.1
			
O1^i^—Co1—N1—C1	−141.42 (12)	N6—C11—C12—C13	0.8 (4)
O1—Co1—N1—C1	38.58 (12)	C11—C12—C13—C14	−1.6 (3)
O2^i^—Co1—N1—C1	127.37 (12)	C11—C12—C13—C18	177.17 (18)
O2—Co1—N1—C1	−52.63 (12)	C12—C13—C14—C15	0.7 (3)
O1^i^—Co1—N1—C5	39.55 (12)	C18—C13—C14—C15	−178.08 (16)
O1—Co1—N1—C5	−140.45 (12)	C11—N6—C15—C14	−1.9 (3)
O2^i^—Co1—N1—C5	−51.66 (12)	C13—C14—C15—N6	1.1 (3)
O2—Co1—N1—C5	128.34 (12)	C20—N7—C16—C17	−1.5 (3)
C5—N1—C1—C2	0.1 (2)	N7—C16—C17—C18	−1.0 (3)
Co1—N1—C1—C2	−179.04 (13)	C16—C17—C18—C19	2.6 (3)
N1—C1—C2—C3	−0.2 (3)	C16—C17—C18—C13	−177.01 (17)
C1—C2—C3—C4	−0.4 (2)	C12—C13—C18—C17	−5.5 (3)
C1—C2—C3—C3^ii^	179.68 (16)	C14—C13—C18—C17	173.20 (17)
C2—C3—C4—C5	1.0 (2)	C12—C13—C18—C19	174.9 (2)
C3^ii^—C3—C4—C5	−179.08 (16)	C14—C13—C18—C19	−6.4 (3)
C1—N1—C5—C4	0.6 (2)	C17—C18—C19—C20	−1.8 (3)
Co1—N1—C5—C4	179.65 (11)	C13—C18—C19—C20	177.9 (2)
C3—C4—C5—N1	−1.1 (2)	C16—N7—C20—C19	2.5 (4)
C15—N6—C11—C12	0.9 (3)	C18—C19—C20—N7	−0.8 (4)

Symmetry codes: (i) −*x*+1, −*y*, −*z*+1; (ii) −*x*+1, −*y*, −*z*.

### Hydrogen-bond geometry (Å, °)

**Table:**

*D*—H···*A*	*D*—H	H···*A*	*D*···*A*	*D*—H···*A*
O1—H1A···O1W	0.86 (2)	1.89 (2)	2.7406 (19)	176 (2)
O1—H1B···N7^iii^	0.85 (2)	1.94 (2)	2.7744 (18)	165 (2)
O1W—H1WA···O3^iv^	0.87 (2)	2.08 (2)	2.924 (2)	164 (2)
O1W—H1WB···O6	0.88 (2)	2.19 (2)	3.070 (3)	174 (2)
O2—H2A···N6^v^	0.88 (2)	1.83 (2)	2.7058 (19)	174 (2)
O2—H2B···O3^vi^	0.85 (2)	2.21 (2)	2.957 (2)	147 (2)

Symmetry codes: (iii) −*x*+1, −*y*+1, −*z*; (iv) −*x*, −*y*+1, −*z*+1; (v) *x*+1, *y*, *z*; (vi) *x*+1, *y*−1, *z*.
